# Transdiscal Fixation for Dropped Head Syndrome: A Case Report

**DOI:** 10.7759/cureus.78947

**Published:** 2025-02-13

**Authors:** Masato Tanaka, Ahmed Heydar, Muhamd A Rahmen, Tadashi Komatsubara, Shinya Arataki

**Affiliations:** 1 Orthopedic Surgery, Okayama Rosai Hospital, Okayama, JPN; 2 Orthopedic Surgery, Okayama University Hospital, Okayama, JPN; 3 Orthopedics and Traumatology, Bahçelievler Memorial Hospital, Istanbul, TUR

**Keywords:** adult spinal deformity, distal junctional kyphosis, drop head syndrome, surgery, transdiscal screw

## Abstract

Dropped head syndrome (DHS) is a rare condition mainly due to neck extensor muscle weakness. The main symptoms of DHS are chin-on-chest deformity, difficulty raising the head against gravity, neck pain, difficulty eating, and difficulty maintaining horizontal gaze. The DHS patients with severe daily life disturbances need surgical intervention, which is usually a long spinal fusion. There are several reports of distal junctional failure due to distal screw loosening, screw pullout, and implant failure because DHS patients are relatively old and may have osteoporosis. To solve this problem, cement-augmented screw fixation is one option. However, due to shoulder shadow, the cervicothoracic junction is complicated to get a clear C-arm image. The report presents the case of a 77-year-old male patient with DHS treated with a novel C-arm-free navigation technique via transdiscal fixation.

## Introduction

Dropped head syndrome (DHS) is a rare condition of neck extensor muscle weakness that causes difficulty in forward gazing due to a chin-on-chest deformity in the standing or sitting position [1]. Symptoms of DHS include neck pain, difficulty lifting the head, swallowing, speaking, and walking. DHS is classified into two pathologies: neuromuscular disorders, such as myasthenia gravis [2] and amyotrophic lateral sclerosis [3], and non-neuromuscular disorders, such as cervical spondylosis, spondylodiscitis, and substance abuse [4-6].

According to radiologic features, DHS has been classified into two categories: sagittal vertical axis (SVA) (−) and SVA (+) [7]. DHS patients with SVA (-) compensate for the sagittal alignment as thoracic kyphosis decreases, lumbar lordosis increases, and the pelvis undergoes retroversion. However, DHS patients with SVA (+) usually have insufficient compensating ability because of increased thoracic kyphosis due to osteoporotic vertebral fracture and/or diffuse idiopathic skeletal hyperostosis and loss of lumbar lordosis due to lumbar spinal stenosis [7].

A cervical collar and neck muscle exercise might be effective for DHS patients with mild symptoms. DHS patients with severe symptoms need surgery, sometimes relatively long spinal fusion from C2 to the mid-thoracic region [8,9]. For osteoporotic vertebrae, regular pedicle screws are not so effective, and distal screws back out may cause distal junctional kyphosis (DJK). Previous reports showed difficulty handling DJK [10,11].

Nowadays, augmented screws are available to prevent screw backout or DJK. At the proximal thoracic level, visualization with the C-arm is poor, and it is challenging to use augmented screws. On the other hand, a transdiscal screw using the navigation provides strong fixation and suits C-arm- free technique. Therefore, the authors present a novel method to prevent DJK from utilizing the strong transdiscal screw at the lower instrumented vertebra during DHS surgery.

## Case presentation

Patient history

A 77-year-old man was referred to our emergency department in April of 2022 with severe neck pain and difficulty in forward gazing. The patient had a T12 osteoporotic vertebral fracture (lumbar BMD T previous score=-1.8 SD) and underwent surgery for balloon kyphoplasty in October 2023. He visited the neurological department and checked for motor neuron disease, but he could not get a definite diagnosis. The five months of conservative treatments, such as neck muscle exercises and cervical collar, were ineffective for him.

Physical examination

The patient had intermediate neck pain and difficulty in forward gazing due to a chin-on-chest deformity. He had slight lower back pain due to a previous osteoporotic vertebral fracture (OVF), and his Oswestry disability index (ODI) was 13. There was no urinary or bowel disturbance. He had slight hyperreflexia on the bilateral leg and could not elevate his left arm due to deltoid muscle weakness (MMT 2). However, he showed no motor deficits in his legs or sensory disturbance. He had no symptoms such as tremors or stiffness, which are suspicious of Parkinson's disease.

Preoperative imaging

**Figure 1:**
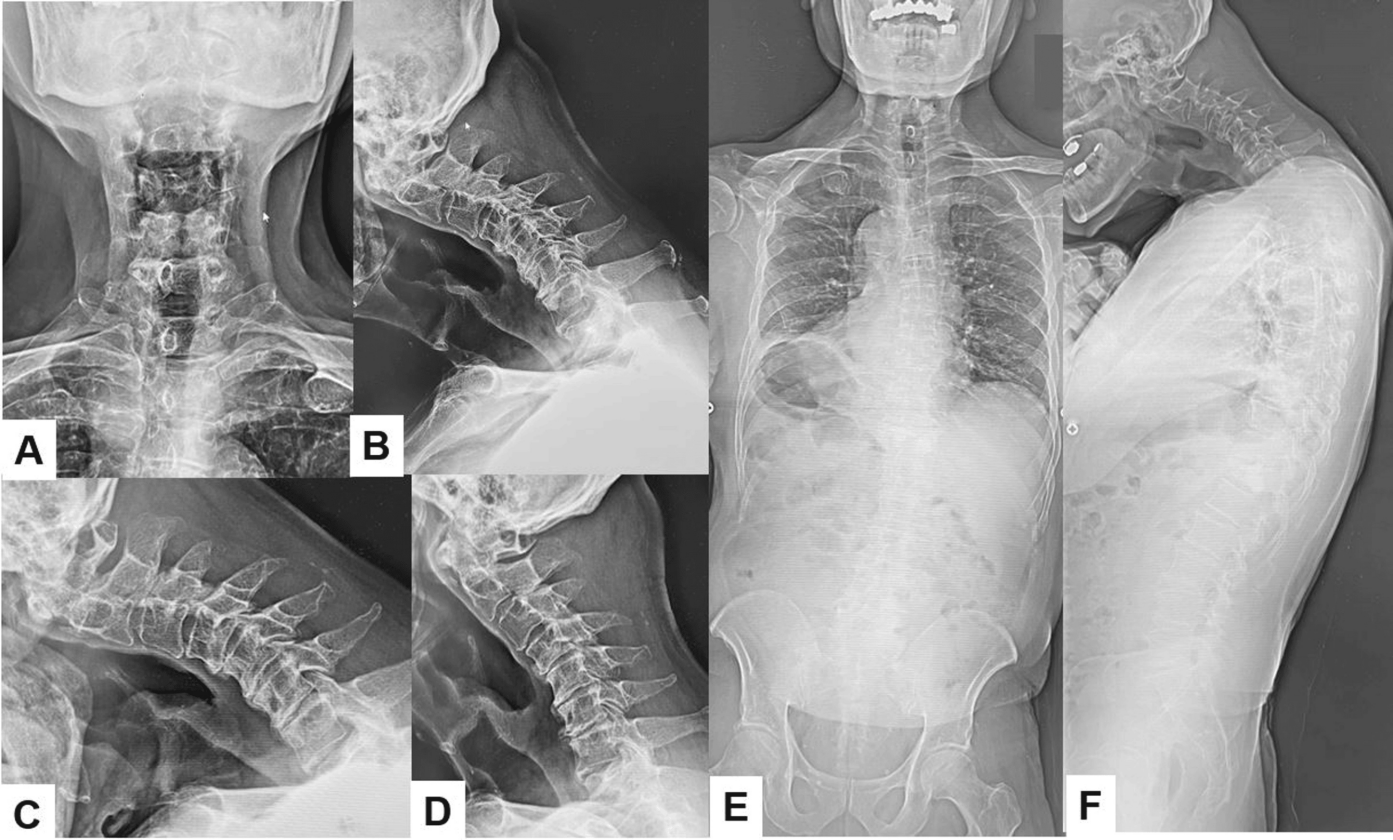
Preoperative radiogram. A. Anteroposterior cervical radiogram. B. Lateral cervical neutral radiogram. C. Lateral cervical flexion radiogram. D. Lateral cervical extension radiogram. E. Posteroanterior standing whole spine radiogram. F. Lateral standing whole spine radiogram. The spinopelvic parameters were as follows: OC2 angle 35, C2-7 kyphosis 36, T1 slop 85, cervical SVA 18.4 mm, PT 42, PI 49, lumbar lordosis 39, T1-4 angle 27, and T4-12 angle 42.

A preoperative cervical radiogram showed severe kyphosis and slight cervical spondylosis (Figures 1B, 1F). Cervical flexion and extension radiograms indicated that his cervical kyphosis was not rigid (Figures 1C, 1D). Cervical parameters were as follows: occtipitoaxial (OC2) angle 35, C2-7 kyphosis 36, T1 slop 85 (Figures 1B, 1F). Standing total spinal radiogram indicated cervical SVA 18.4 mm, pelvic tilt (PT) 42, pelvic incidence (PI) 49, lumbar lordosis 39, T1-4 angle 27, and T4-12 angle 42 (Figure 1F).

**Figure 2:**
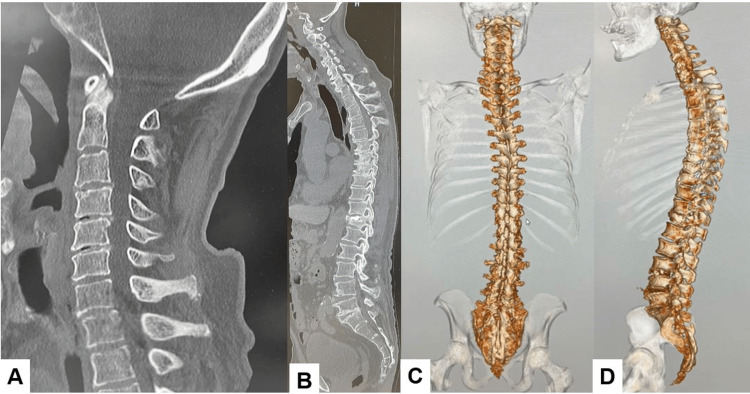
Preoperative CT. A. Cervical mid-sagittal reconstruction CT. B. Whole spine posterior 3D CT. D. Whole spine lateral 3D CT.

Preoperative CT showed collapse of disc spaces in C3-7, but cervical deformity was not rigid. A previous balloon kyphoplasty was performed on the T12 vertebra (Figure 2).

**Figure 3:**
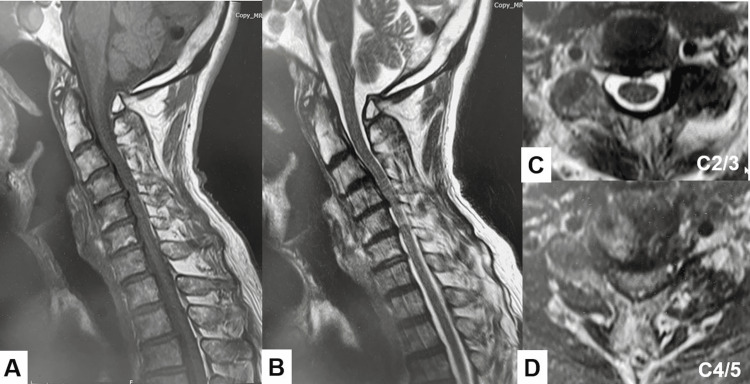
Preoperative MRI. A. T1-weighted mid-sagittal image. B. T2-weighted mid-sagittal image. C. T2-weighted axial image at C2/3. D. T2-weighted axial image at C2/3. The spinal cord was severely compressed at the C4/5 level.

MRI revealed cervical cord compressed at the C3/4, C4/5, and C5/6 levels (Figure 3).

Surgical strategy for DHS

Our surgical strategy for DHS is as follows. The first step is to check the flexibility of cervical kyphosis in the extension position. If the kyphosis is flexible, only a posterior approach is indicated; if the deformity is relatively rigid, anterior and posterior combined surgery is indicated. The second step is to check whether facet joints are mobile or fused. If the facet joints are fused, three-staged surgery (posterior-anterior-posterior) is necessary. If not, then two-staged surgery (anterior-posterior) is enough. The third step is to evaluate cervical canal stenosis or myelopathy/radiculopathy. If the patient has myelopathy/radiculopathy or canal stenosis, laminoplasty, laminectomy, or foraminotomy should be added.

Surgical technique

**Figure 4:**
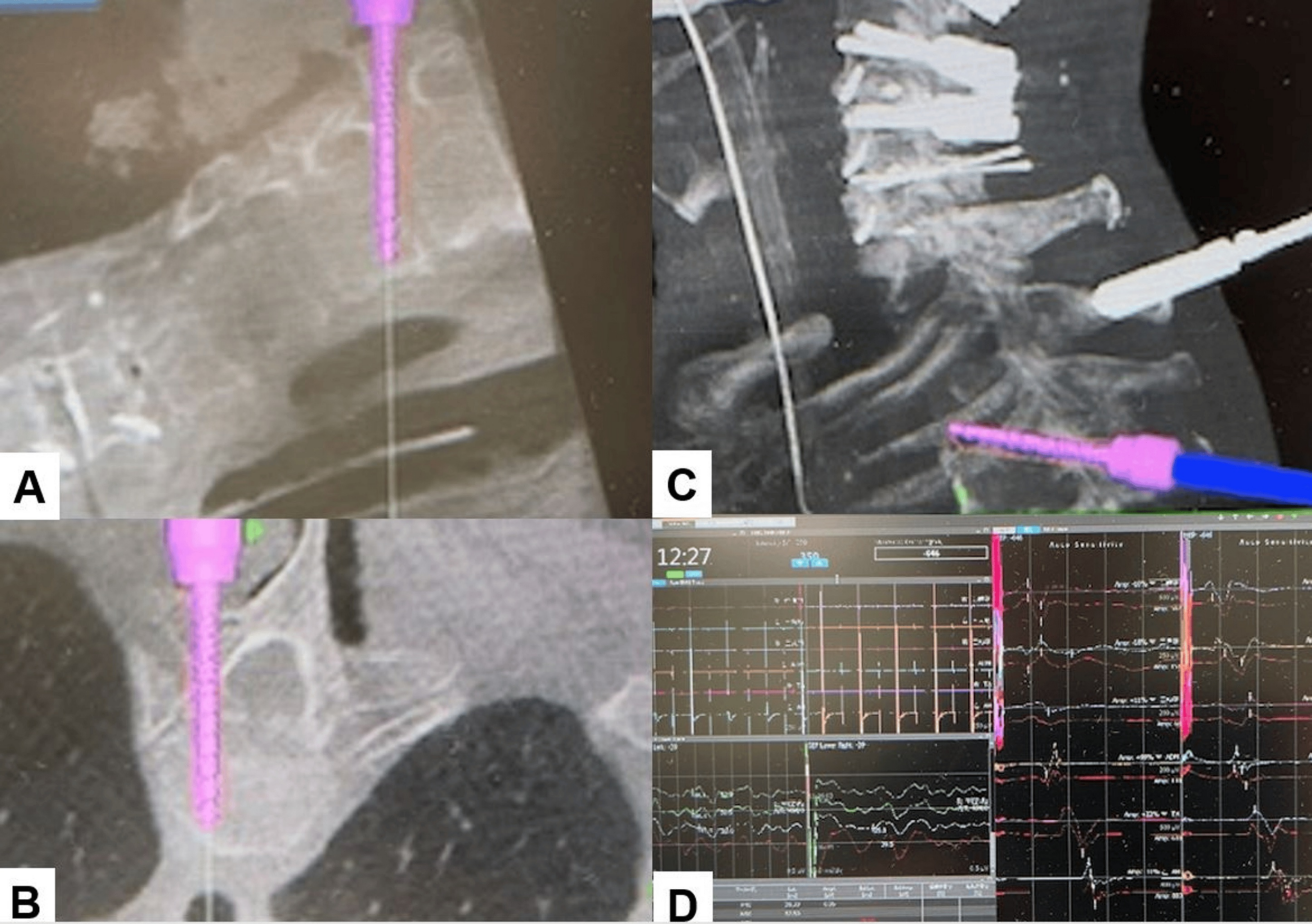
Navigation monitor and neuromonitoring. A. Sagittal image. B. Axial image. C. 3D image. D. Neuromonitoring.

First, the patient was positioned in a supine. Anterior cervical discectomy and fusion (ACDF) were performed at the C45, C5/6, and C6/7 levels to secure the bony fusion and create adequate cervical lordosis. Then, the patient was placed in a prone position on a radiolucent table with a Mayfield skull cramp. Neuromonitoring was performed to prevent intraoperative deterioration of the patient's neurological status. The reference frame for the navigation is fixed at C2/T1 spinous process, the O-arm is positioned, and the intraoperative CT images are obtained and transmitted to the navigation system. Cervical pedicle screws and T2/T3 transdiscal screws were inserted by navigation. Strong anchors such as pedicle screws and transdiscal screws are preferable for the osteoporotic spine to prevent screw backout. The transdiscal screws should be aimed toward the upper endplate to penetrate the superior endplate of the working level (approximately 25 degrees) (Figure 4).

**Figure 5:**
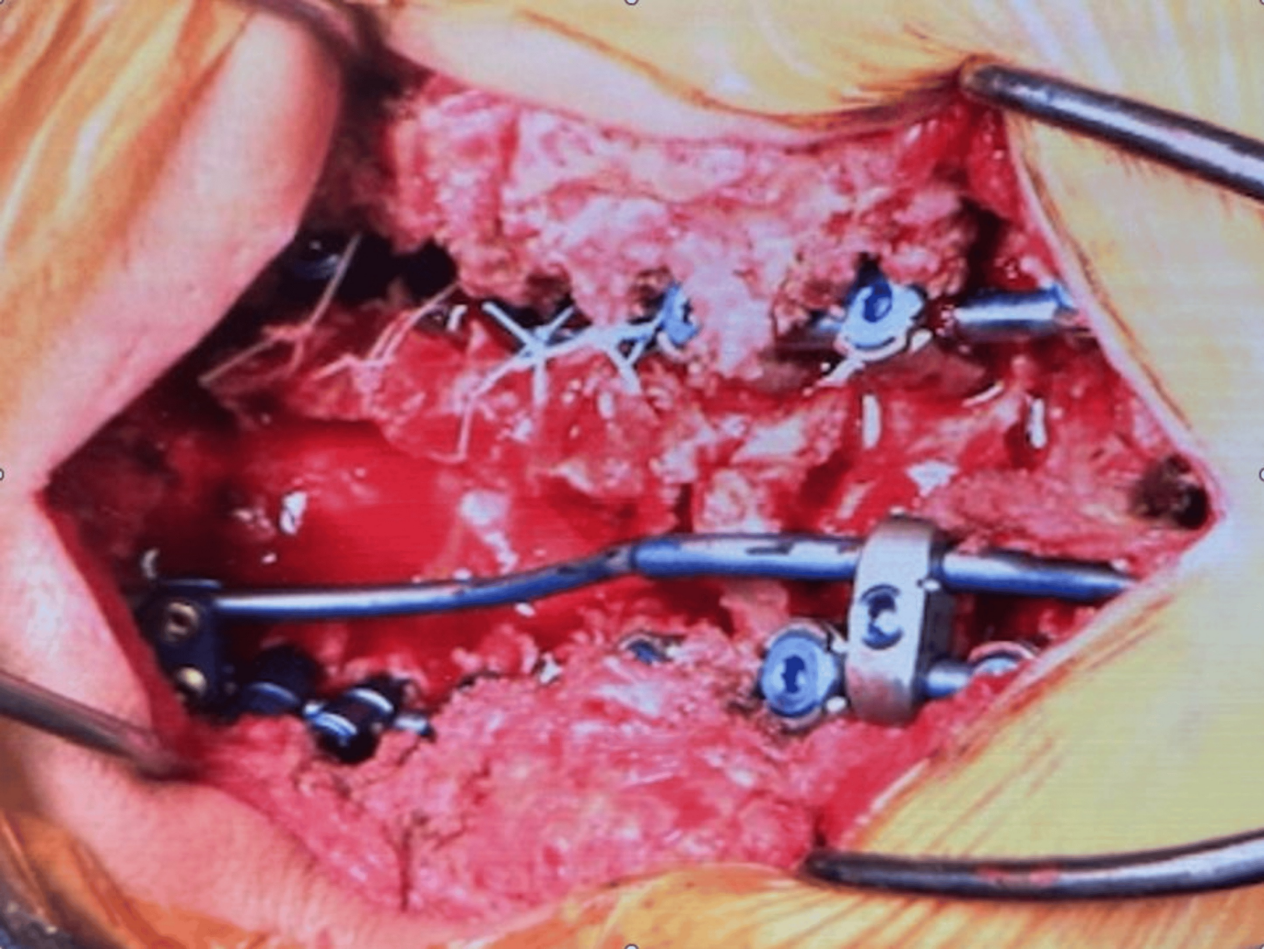
Intraoperative image. Three rods were applied to prevent rod breakage.

**Figure 6:**
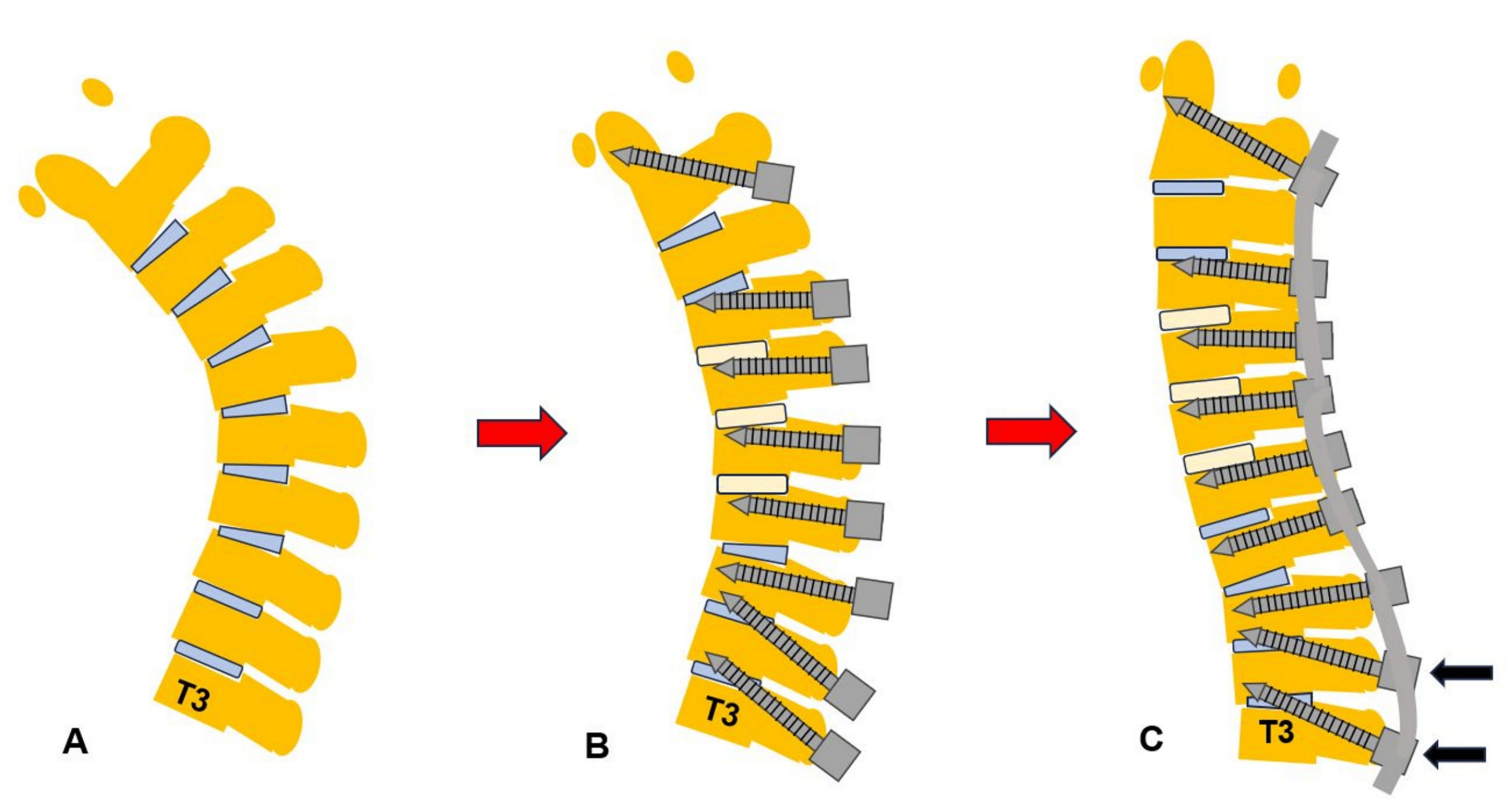
Shema of the novel technique for DHS. A. Preoperative image. B. Three-level anterior cervical discectomy and fusion. C. Grade 2 osteotomy and corrective fusion. Black arrows indicate transdiscal screws.

After that, grade 2 posterior osteotomies were performed to correct the cervical kyphosis. Finally, approximately three contoured rods were applied, and adequate cervical lordosis was created. C3 laminectomy, C4-7 laminoplasty, and right-side posterior bone grafting were completed (Figures 5, 6). Surgical time was 6 hours and 48 minutes, and blood loss was 190 mL.

Postoperative images

**Figure 7:**
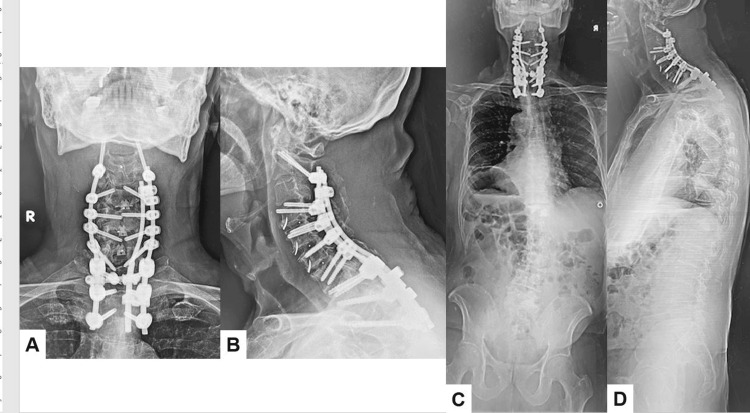
Postoperative images. A. Anteroposterior cervical radiogram. B. Lateral cervical radiogram. C. Posteroanterior whole spine radiogram. D. Lateral whole spine radiogram. Excellent spinal alignment was obtained.

Postoperative spinal radiograms showed good cervical alignment. In a lateral cervical radiogram, T2/T3 transducer screw position is adequate, and postoperative CT indicated all screw positions were satisfactory (Figure 7).

Clinical results

**Figure 8:**
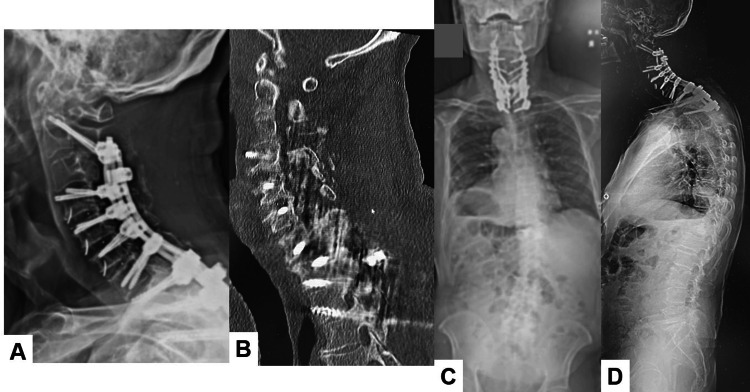
Final follow-up images. A. Cervical lateral radiogram. B. Mid sagittal cervical CT. C. Posteroanterior whole spine radiogram. D. Lateral whole spine radiogram. The spinopelvic parameters were as follows: OC2 angle 23, C2-7 kyphosis 26, T1 slop 45, cervical SVA 49.5 mm, PT 39, PI 49, lumbar lordosis 39, T1-4 angle 32, and T4-12 angle 33.

Five days after surgery, he could walk with a cervical collar. He had no neurological deficit, and he could see straight forward. His deltoid muscle weakness was partially recovered. Solid cervical fusion was obtained seven months after surgery, and global spinal alignment was acceptable (Figure 8).

.

## Discussion

In the present study, we report a case of a patient with DHS who underwent surgical intervention utilizing a novel transdiscal posterior instrumentation technique in conjunction with ACDF. Cervical spinal cord decompression was accomplished through C3 laminectomy and C4-7 open-door laminoplasty. Favorable outcomes regarding deformity correction and neurological improvement were observed during short-term follow-up.

While conservative treatment is typically the initial approach for DHS [12], it is primarily considered for patients with severe comorbidities and is restricted to strengthening exercises and collar usage. Despite their ability to keep the head upright, cervical collars are often poorly tolerated by patients and may cause pressure sores under the chin and occiput. A theoretical disadvantage is that collar use might further weaken neck muscles as patients rely on the collar for support [12,13]. Furthermore, only a few patients have shown improvement following non-surgical treatment [14]. Conversely, multiple studies have reported positive outcomes from surgical treatment for DHS [9]. However, due to limited information on surgical interventions, there is no clear agreement on the best approach [15]. Moreover, various mechanical and neurological complications associated with surgical treatment have been documented [8,9].

The surgical approach, whether posterior-only or combined anteroposterior, the occiput in the fusion segments, and the number of levels to be included in the construct are subjects of controversy in the surgical treatment of DHS [16]. Although the surgical approach should be individualized for each patient, we generally prefer a combined anteroposterior approach in our clinical practice, exclude the occiput and C1 from the fusion segments, and extend the construct to the upper thoracic spine. These measures are implemented to retain some degree of rotation and flexion-extension movement at the proximal cervical junction, achieve better deformity correction, ensure thorough cord decompression, increase biomechanical support, and reduce failure risks. Our strategy aligns with those of other researchers [13,16].

Bones affected by osteoporosis are considered significant factors contributing to implant failure or inadequate fixation [1] and are also associated with the occurrence of DJK following surgical treatment of DHS [8]. Research by Yagi et al. [17] indicated that teriparatide administration reduced the incidence of bone failure-related proximal junctional kyphosis after adult spinal deformity surgery. Furthermore, prophylactic use of parathyroid hormone was suggested to mitigate the risk of distal junctional kyphosis in osteoporotic patients undergoing surgical correction of DHS [8].

At the proximal thoracic level, visualization with the C-arm is poor, and it is challenging to use augmented screws. On the other hand, a transdiscal screw using the navigation provides strong fixation and suits C-arm- free technique. Therefore, the authors present a novel method to prevent DJK from utilizing the strong transdiscal screw at the lower instrumented vertebra during DHS surgery. With our technique, the surgeon can reduce the fusion level because distal transdiscal screws are powerful anchors for the osteoporotic vertebrae. Biomechanical research has demonstrated that these screws provide 1.6 to 1.8 times greater fixation strength compared to conventional pedicle fixation [18]. However, the complex trajectory and challenges in obtaining high-quality intraoperative fluoroscopic images limit its widespread adoption. Nevertheless, advancements in intraoperative navigation systems have made transdiscal pedicle placement more feasible [19].

A limitation of the study is the brief follow-up period of the patient, which necessitates long-term observation. However, this research could serve as a foundation for further outcome studies and comparison with results of alternative techniques, as the employed surgical method represents a viable option in treating similar cases, with the potential to mitigate mechanical failure. This technique has potential risks, such as spinal cord injury or screw perforation to the vertebral artery. Navigation is preferable for this technique.

## Conclusions

DHS and osteoporosis present complex challenges for spine surgeons. With our technique, the surgeon can reduce the fusion level because distal transdiscal screws are powerful anchors for the osteoporotic vertebrae. This technique can be performed under navigation guidance without C-arm usage. Furthermore, lateral images of the C-arm in the cervicothoracic junction are challenging to get clear images. The C-arm-free technique is beneficial for spine surgeons and the operation staff to prevent radiation hazards.
